# Feasibility of early interval training in patients recovering from heart valve surgery due to infective endocarditis

**DOI:** 10.1186/s40814-026-01830-w

**Published:** 2026-05-12

**Authors:** Margrethe Müller, Tove Aminda Hanssen, David Johansen, Øyvind Jakobsen, John Erling Pedersen, Inger Lise Aamot Aksetøy, Trine Bernholdt Rasmussen, Gunnar Hartvigsen, Vegard Skogen, André Henriksen, Gyrd Thrane

**Affiliations:** 1 Department of Physiotherapy, University Hospital of North Norway, Tromsø, Norway; 2 Department of Health and Care Sciences, UiT The Arctic University of Norway, Tromsø, Norway; 3 Department of Cardiology, University Hospital of North Norway, Tromsø, Norway; 4 Department of Clinical Medicine, UiT The Arctic University of Norway, Tromsø, Norway; 5 Department of Internal Medicine, University Hospital of North Norway, Tromsø, Norway; 6 Department of Cardiothoracic and Vascular Surgery, University Hospital of North Norway, Tromsø, Norway; 7 Norwegian University of Science and Technology, Trondheim, Norway; 8 Clinic of Rehabilitation, St Olavs Hospital, Trondheim University Hospital, Trondheim, Norway; 9 Department of Cardiology, Herlev and Gentofte University Hospital, Hellerup, Denmark; 10 Department of Clinical Medicine, University of Copenhagen, Copenhagen, Denmark; 11 Department of Computer Science, UiT The Artic University of Norway, Tromsø, Norway; 12 Helgeland Hospital Trust, Sandnessjøen, Norway

**Keywords:** Aerobic interval training, Heart valve surgery, Infective endocarditis, Rehabilitation

## Abstract

**Background:**

Infective endocarditis is an infectious heart disease strongly associated with morbidity and mortality. Up to half of the patients with infective endocarditis require heart valve surgery. While early exercise-based rehabilitation is well documented for patients recovering from heart surgery for non-infective endocarditis, there is limited research on those who have undergone valve surgery due to this infection. This study aimed to explore the early aerobic training in this patient population.

**Methods:**

A single-centre prospective feasibility study was conducted using the UK Medical Research Council’s framework for complex interventions. The study investigated the feasibility (recruitment, retention, adherence), safety, acceptability, and preliminary functional outcomes of 4 × 4 interval training in this patient population. Training session data included the number, duration, and intensity, which were monitored via the Apple Watch S5 (Present Age-Predicted Maximum Heart Rate) and the Borg RPE scale. Functional outcomes were evaluated at baseline and 3 months post-surgery, including sub-maximal oxygen uptake (treadmill protocol), 6-min walk test, and quality of life (HeartQoL, EQ-5D-5L).

**Results:**

Sixteen patients consented to participate, with 12 initiating the intervention and 11 completing it, yielding a retention rate of 91.7%. Training adherence averaged 73.1% of the minimum expected sessions, with high participant satisfaction and no serious adverse events reported. At the 12-week follow-up, participants demonstrated measurable change in functional capacity, including an increase in workload capacity (+ 95 W), METs (+ 3.4), and 6-min walk test distance (+ 219 m). Health-related quality of life also showed a noticeable increase, with HeartQoL physical and emotional scores increasing by 1.0 and 1.3, respectively, and EQ-5D-5L VAS scores rising by 17.2. The EQ-5D-5L index increased from 0.61 at baseline to 0.87 after 12 weeks.

**Conclusion:**

Interval training, when conducted with appropriate safeguards and tailored to individual needs, is a feasible and safe intervention for patients recovering from endocarditis and cardiac surgery. The observed improvements in functional capacity, quality of life, and patient satisfaction support the need for larger controlled studies.

**Trial registration:**

Clinical Trials, ID NCT05703022. Registered on 25 November 2021, http://www.ClinicalTrials.gov

**Supplementary Information:**

The online version contains supplementary material available at 10.1186/s40814-026-01830-w.

## Key messages regarding the feasibility

- What uncertainties existed regarding the feasibility?
  Limited research exists on the feasibility of interval training in patients recovering from heart valve surgery due to infective endocarditis.
  The safety and the acceptability of early postoperative interval training in this patient group remain unknown.
- What are the key feasibility findings?
  This study addresses a critical gap in the literature by evaluating rehabilitation strategies for patients recovering from heart valve surgery due to infective endocarditis.
  The findings demonstrate that interval training using the modified 4x4 method is feasible with respect to recruitment, retention, and adherence among patients with postoperative endocarditis and was well tolerated and acceptable.
- What are the implications of the feasibility findings for the design of the main study?
  These findings offer valuable insights to inform and enhance the structure of future studies. They highlight the importance of training-based monitoring and inform the design of the main study by refining inclusion criteria, optimising training protocols, and ensuring patient safety and adherence.

## Introduction

Infective endocarditis (IE) is a serious infectious heart disease that, although rare, is strongly associated with morbidity and mortality [1–3]. These patients often experience a decline in physical fitness and endurance [4–6]. Approximately one-third report a sedentary lifestyle with poor physical and mental health, while two-thirds fail to achieve adequate levels of daily functioning [4, 7, 8]. A feasibility study on aerobic interval training (AIT) following cardiac surgery suggests that AIT is not only feasible but also practical and safe, with improvements in walking function and maximal oxygen uptake (VO₂max) [9, 10]. Training-based cardiac rehabilitation enhances cardiovascular fitness and reduces the risk of recurrence and mortality in patients with cardiovascular disease. The safety of interval training for patients with heart disease is well supported, with a serious adverse event rate of just 1 per 23,182 h of training [11]. Among available training models, the 4 × 4 interval training protocol is well established and widely used [12–14]. This method involves four intervals of 4 min at high intensity, interspersed with active recovery periods, and is considered the ‘gold standard’ for cardiac rehabilitation in Norway [15]. Its flexibility allows for individual adaptation according to clinical status and physical capacity. This makes it particularly suitable for the present study population with infective endocarditis undergoing heart valve surgery, who often present with postoperative complications, multiple comorbidities, and variable physical impairments. Despite advantages in post-surgical recovery, insufficient evidence in the IE patient group prevents the establishment of departmental and international guidelines, which limits the justification for investment in exercise-based rehabilitation programmes. 

Several distinctions between patients with IE after valve surgery and those without IE conditions highlight the need for tailored exercise-based rehabilitation. IE treatment is complex and requires interdisciplinary teams of various specialists and physiotherapists, high-dosage antibiotics, and heart valve surgery in up to 50% of cases [16, 17]. Patients with IE who undergo valve surgery are notably more debilitated, have an increased risk of postoperative atrial fibrillation, and frequently exhibit greater heterogeneity in comorbidities and age [17, 18]. Because the postoperative course is complicated by arrhythmias, concurrent cardiac rhythm monitoring is important [18]. Our previous research demonstrated that single-lead smartwatch ECG detection of atrial fibrillation was comparable to continuous telemetry monitoring, with a high accuracy for detecting AF [19].

Insights gained from cardiac surgery patients without IE may not directly apply to those with IE. There is a lack of knowledge regarding the feasibility, safety, and acceptability of such interventions in postoperative endocarditis patients.

The study aimed to evaluate the feasibility of an early exercise intervention for patients following heart valve surgery and endocarditis, focusing on recruitment, retention, adherence, safety, acceptability, and preliminary functional outcomes.

## Materials and methods

### Study design

This study was designed as a quantitative feasibility study within the UK Medical Research Council (MRC) framework, using multiple quantitative measures to assess feasibility, safety, acceptability, and preliminary functional outcomes [20, 21].

Feasibility was defined as the extent to which the intervention and study procedures could be successfully delivered and was quantified through recruitment, retention, and adherence.

Additionally, safety and acceptability were considered integral components, as an intervention that is unsafe or unacceptable cannot be successfully implemented.

Accordingly, the following outcomes were evaluated:

- Acceptability, defined as participants’ perceptions of the appropriateness, burden, and satisfaction of the intervention; and
- Safety, defined as the occurrence of intervention-related adverse events.

Patient involvement: Two patient representatives were involved throughout the project: one patient with infective endocarditis who had undergone valve surgery and one patient with established heart disease. Their contributions informed the study design and conduct of the study.

### Inclusion and exclusion criteria

Participants were eligible for inclusion if they were aged ≥ 18 years, had confirmed infectious endocarditis with left-sided valve surgery without arterial embolus, were residents of Northern Norway, and were willing and able to provide informed consent between 4 and 21 days postoperatively.

Exclusion criteria included hemodynamic or respiratory instability, body temperature > 38 °C or positive blood cultures at the time of assessment, clinically significant severe comorbid medical conditions or active serious infections affecting safety or tolerability of the intervention, and musculoskeletal or other conditions that could limit participation in physical activity.

### Study setting and recruitment

**Fig. 1:**
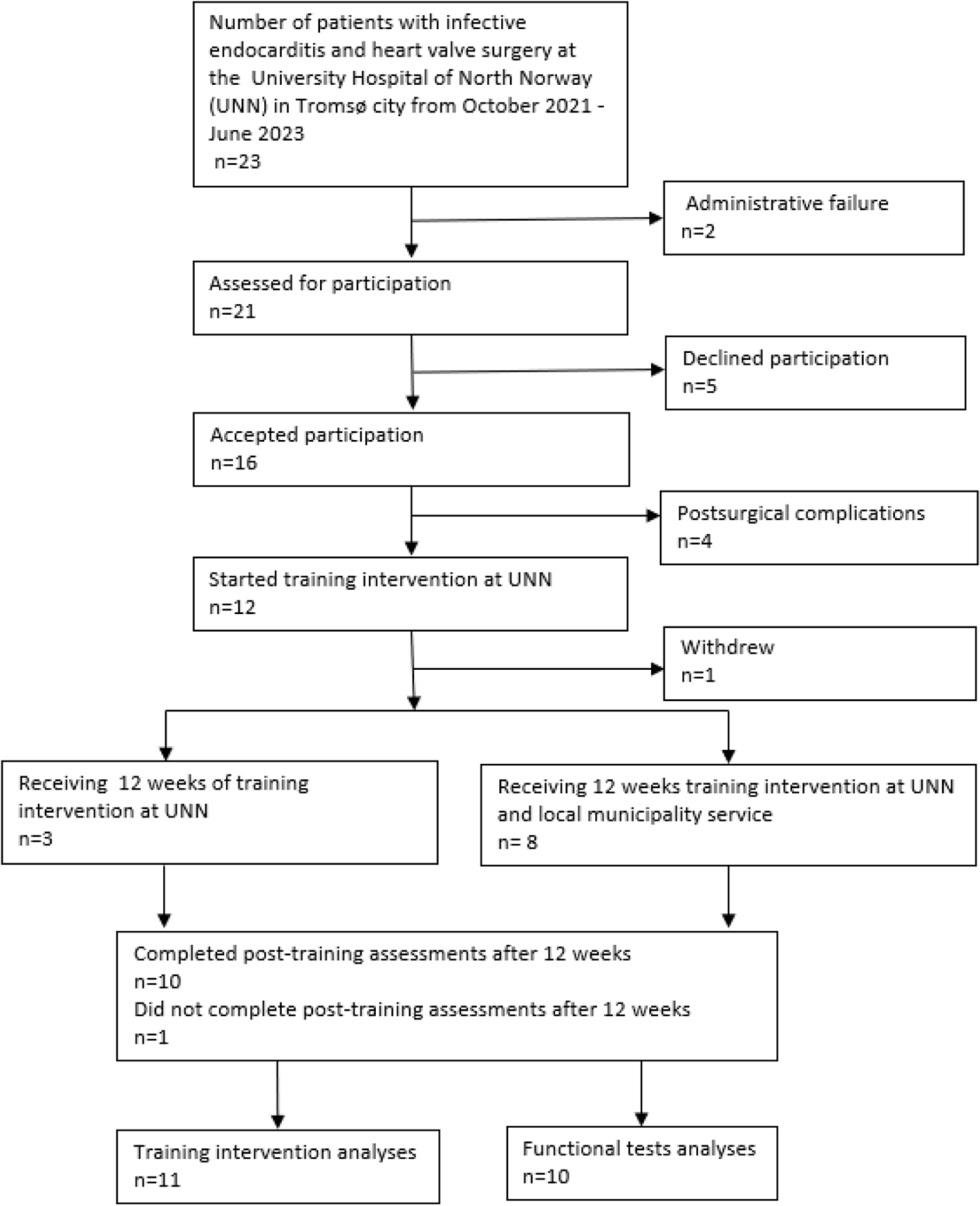
An outline of patient recruitment, screening, intervention, and data collection

Between October 2021 and June 2023, 23 consecutive patients who underwent valve surgery for IE were identified from the patient administrative system at the University Hospital of North Norway (UNN). Of these, 21 participants who met the inclusion criteria were informed about the study and invited to participate. Figure 1 illustrates the study setting and recruitment process.

### The early aerobic interval training intervention

Participants completed a supervised interval training programme based on the 4 × 4 interval model. Each session consisted of four 4-min intervals of high intensity, interspersed with active recovery periods. The duration of the intervals was adjusted based on individual clinical status and physical capacity.

All participants received standard postoperative beta-blocker therapy. Heart rate was monitored during exercise using a smartwatch with ECG functionality. The intervention was initiated between 7 and 36 days after surgery when the participants were hemodynamically and respiratory stable, had a temperature below 38 °C, and had a set of negative blood cultures. Both during hospitalisation and after discharge, subjects participated in supervised aerobic interval training for 30 to 40 min, 2 to 3 times a week. Exercise equipment (e.g. treadmill, indoor bicycle) was selected based on the physiotherapist’s assessment, available equipment, and participant preference. Exercise sessions were discontinued if signs of functional deterioration were observed, including dyspnoea, fever, arrhythmia, or worsening heart failure.

### Training details

Each session included:

- Warm-up: 4–10 min
- Interval training: 8–16 min of high-intensity bouts (2–4 min each, repeated 4 times), starting at 60% of maximum heart rate and gradually increasing up to 85%, with a perceived exertion of 13–17 Borg Rating of Perceived Exertion Scale (RPE) [22].
- Recovery periods: 2–4 min between high-intensity bouts, at 40% of maximum heart rate.
- Cooldown: 4–6 min

The smartwatch's ECG function was used after each exercise to monitor heart rate and rhythm activity. C-reactive protein (CRP) levels and body temperature were measured before each interval training session, while blood pressure was measured both before and after each session.

### Data collection and analysis

#### Feasibility outcomes

Feasibility was defined as the extent to which the intervention and study procedures could be successfully delivered. In this study, feasibility was assessed using three predefined parameters: recruitment, retention, and adherence.

Predefined progression criteria for recruitment, retention, and adherence were set a priori based on methodological recommendations for feasibility studies calling for clear quantitative benchmarks (≥ 50% recruitment and ≥ 60–70% retention thresholds and ≥ 70% of adherence—expected training sessions were completed) to assess feasibility. These criteria are similar to those used in other feasibility studies, where a 50% recruitment rate and retention rates of ≥ 70% have been used to judge study feasibility [23]. To monitor adherence, we collected detailed data on the number, duration, and frequency of training sessions, exercise intensity, training equipment, and methods used. During treadmill interval training, speed and incline for each interval were recorded.

#### Acceptability outcomes

Acceptability specifically referred to the intervention experience with training components, session structure, collaboration with a physiotherapist, and smartwatch usability.

Participants' experiences with the training components and the overall intervention were assessed using a specifically developed questionnaire that included 24 items: 17 items were graded on a five-point Likert scale (from ‘not at all’ to ‘to a very great extent’), and four items were rated on a three-point bipolar scale. The five-point Likert scale items include: ‘To what extent did you feel confident in training?’ and ‘Overall, how satisfied are you with the training offered after surgery?’. Examples of the three-point bipolar scale items include: ‘Do you think that the initiation of training after surgery occurred?’ with response options ‘too early’, ‘appropriate’, and ‘too late’ and ‘Do you think that the level of exertion during training was’ with response options ‘too low’, ‘appropriate’ and ‘too high’. Three open-ended questions: ‘What could have been improved with the training offer?’ ‘What do you think was good about the training offer?’ and ‘Do you have any other suggestions/comments about the training offer?’ The full questionnaire is in the appendix, including all 24 items and their response options.

#### Functional outcomes

Sub-maximal oxygen uptake was measured at baseline and again at 12 weeks post-surgery using a standardised ramp protocol on a treadmill, monitored by the MasterScreen CPX spirometry system with Jaeger Lab Software [24]. The stress test lasted between 8 and 12 min and followed the Prozac protocol with 30-s intervals and a 0.5 MET increase at each stage. Walking capacity was assessed at both time points using the 6-min walk test [25, 26]. Quality of life was evaluated using the HeartQoL questionnaire, a disease-specific tool consisting of 14 items assessing physical and emotional dimensions of relevance to heart disease patients [27]. This questionnaire was administered at baseline and 3 months post-surgery, providing a global score alongside physical and emotional subscale scores. The HeartQoL has been validated in Norway, and it is reliable and responsive in patients with various cardiovascular conditions, including those following heart valve surgery [27, 28]. Additionally, the EQ-5D-5L questionnaire, a generic and validated patient-reported outcome measure, was used to assess overall health-related quality of life [29].

#### Sample size justification

This study was designed as a feasibility study, with the primary aim of assessing the feasibility of delivering early interval training to patients recovering from infective endocarditis.

Based on recommendations in the feasibility literature, feasibility studies typically include 12–30 participants, which is considered sufficient to estimate feasibility parameters with adequate precision to inform planning of a full-scale trial. Reviews of pilot and feasibility trials report median sample sizes of around 30 participants (IQR 20–50), with many using pragmatic rules of thumb such as 12 participants per arm [30, 31]. Consequently, we targeted 12–20 participants to generate preliminary estimates of recruitment, retention, adherence, and safety that could inform the design and sample size of a future full-scale trial.

### Statistical analysis

Analyses were primarily descriptive and exploratory, in line with the specified scoring instructions of the instruments and the standard recommendations for feasibility studies [20]. Descriptive statistics were used to summarise participant characteristics, implementation outcomes, and changes in functional and health-related quality of life measures over time. Continuous variables are reported as means with standard deviations (SD), and mean changes from baseline to follow-up are presented with corresponding 95% confidence intervals (CI). Normality of distributions was assessed through visual inspection of histograms and Q-Q plots. Analyses were conducted using SPSS version 29.0.1.0 (IBM Corp., Armonk, NY).

### Ethics and consent

The study was approved by the Regional Committees for Medical and Health Research Ethics in Norway (Project number 228905) and by the Data Protection Authority at the University of North Norway (Project number: 02752) and was registered in ClinicalTrials.gov (NCT05703022). All participants provided both oral and written consent before surgery and study initiation. This study is reported by the guidance Criteria for Reporting the Development and Evaluation of Complex Interventions in Healthcare (CReDECI 2) [21].

## Results

### Feasibility outcomes

#### Recruitment and retention rates

A total of 23 patients were identified, and 21 were invited to participate in the study. Of these, 16 provided informed consent, resulting in a recruitment rate of 69.6%. Four participants did not initiate the intervention after providing informed consent. Two tested positive for COVID-19 postoperatively and were transferred to an isolation unit, which prevented study participation. One was diagnosed with advanced malignancy and transferred to the oncology department for further treatment. Another died due to postoperative complications before commencement of the intervention. Twelve patients initiated the intervention, but one withdrew due to a pre-existing back pain that was unrelated to the exercise programme but exacerbated by postoperative recovery and required medical management. Of the remaining participants, 11 completed the intervention, yielding a retention rate of 91.7%. At the 12-week follow-up, 10 participants underwent functional test assessments. The study population consisted of a greater proportion of men than women and had a broad age range, with participants ranging in age from 41 to 80 years. Additionally, the participants represented various body mass index (BMI) values and varied physical activity levels, from sedentary to highly active individuals. Most participants were cohabiting or married during the study, reflecting a social profile commonly seen in the general population. All patients initiated the intervention at the University Hospital of North Norway (UNN), where supervised training sessions were conducted individually by the study physiotherapist (first author). Patients from Tromsø City (*n* = 3) completed all sessions on-site at UNN. Patients from rural areas (*n* = 8) were referred upon hospital discharge to a local physiotherapist at their nearest hospital or rehabilitation centre to continue the 12-week programme. These physiotherapists received both written and verbal instructions regarding the training protocol and use of the smartwatch for heart rate/rhythm monitoring. They also had the opportunity to consult with the study physiotherapist via telephone or video call for guidance. All training sessions were delivered individually; no group sessions were conducted. Participant characteristics are shown in Table 1.

**Table 1:** Patient characteristics

Characteristics	Participants who completed the intervention *n* = 11	Participants who initiated the intervention *n* = 12
Sex, *n* (%)		
Male	7(64)	8(67)
Age		
Median (range)	69.5 (49–80)	70 (49–80)
Works status, *n* (%)		
Working	4 (36.4)	4 (33.6)
Retired or disability benefits	7 (63.6)	8 (66.7)
Level of education, *n* (%)		
Primary schools	2 (18.2)	2 (16.7)
Upper secondary school	7 (63.6)	8 (66.7)
University	2 (18.2)	2 (16.7)
Cohabiting status, *n* (%)		
Living alone	5 (45.5)	5 (41.7)
Cohabiting	6 (54.5)	7 (58.3)
Body mass index, *n* (%)		
0–18.4	1 (9.1)	1 (8.3)
18.5–24.9	3 (27.3)	3 (25.1)
25–29.9	6 (54.5)	7 (58.3)
30–34.9	1 (9.1)	1 (8.3)
Activity level, *n* (%)		
Sedentary	4 (36.4)	5 (41.7)
≥ 4 times/week	5 (45.5)	5 (41.7)
≤ 4 times/week	2 (18.2)	2 (16.6)
Types of endocarditis, *n* (%)		
Prosthetic valve	7 (63.6)	8 (66.7)
Native valve	4 (36.4)	4 (33.3)
Types of surgery, *n* (%)		
BAVR	7 (63.6)	8 (66.7)
MAVR	4 (36.4)	4 (33.3)

*BAVR*, bioprosthetic aortic valve replacement; *MAVR*, mechanical aortic valve replacement

#### Adherence to the intervention

Participants attended an average of 17.6 training sessions during the 12-week intervention period, corresponding to 73.1% of the minimum expected sessions (24 sessions) and 48.7% of the maximum expected sessions (36 sessions). The mean time to initiate the intervention postoperatively was 12.7 days, ranging from 4 to 36 days. At the end of each interval, the average training intensity, expressed as a percentage of age-predicted maximum heart rate (%APHRmax), was 76.6%. The average duration of four interval sessions was 14.8 min, while the entire training session lasted an average of 25.4 min.

#### Safety and adverse events

All participants received beta-blocker treatment with individually adjusted doses throughout the exercise intervention. No serious adverse events were reported during the intervention. Three participants experienced complications or adverse events. One developed post-pericardiotomy syndrome after undergoing a submaximal treadmill test, though it remains unclear if the test acted as a trigger. This individual received specialist care for 1 week before resuming training. Another participant was diagnosed with atrial fibrillation during the second session, necessitating an adjustment in beta-blocker dosage. The third participant sustained a hip injury after slipping on ice, which necessitated a 14-day hiatus from training. Despite these incidents, all three participants completed the 12-week intervention.

#### Acceptability outcomes

All participants reported acceptability with both the training method and training load. Regarding timing of the intervention, 72% reported being satisfied, while 18% reported that training was initiated too late in their postoperative recovery. With respect to programme structure, 81% reported satisfaction with the number of training sessions, and 90% reported the duration of individual sessions to be appropriate.

Concerning smartwatch use, 54.5% reported that immediate heart rate and pulse feedback was beneficial, 18.2% reported a need for additional instruction to use the device effectively, and 27.3% reported limited utility of the smartwatch.

#### Functional outcomes

During the 12-week training intervention, patients experienced improvements in their functional capacity. Table 2 presents the estimates of sub-maximal oxygen uptake, workload capacity, metabolic equivalents (METs), peak heart rate, and 6-min walk distance at baseline and follow-up, along with the corresponding mean changes and 95% confidence intervals.

**Table 2:** Sub-maximal oxygen uptake measured at baseline and 3 months post-surgery using a standardised ramp protocol on a treadmill

	**Pretest ± SD*n* = 12**	**Posttest ± SD* n* = 11**	**MD (95%CI)**
VO₂peak (mL·kg⁻^1^·min⁻^1^)	11.2 ± 2.8	19.1 ± 5.5	7.9 (4.8,10.9)
RERpeak	0.9 ± 0.1	1.1 ± 0.1	0.2 (0.0,0.3)
VEpeak (L/min)	33.8 ± 13.9	60.8 ± 17.9	26.9(10,43.8)
O₂peak (L/min)	7.1 ± 1.9	10.5 ± 3.5	3.4 (1.8,5)

*SD* Standard deviation, *MD* Mean difference, *VO₂peak* Volume of peak oxygen uptake, *RERpeak* Respiratory exchange ratio peak, *VEpeak* Ventilatory equivalent peak, *O₂peak* Peak oxygen level

Workload capacity increased from a mean of 93 ± 65 W at baseline to 188 ± 81 W post-intervention, with a mean change of 95 W (95% CI: 56.6 to 134). METs increased from 4.9 ± 2.9 to 8.3 ± 3.4. Peak heart rate rose from 124.3 ± 22.2 bpm to 141.2 ± 18.7 bpm, with a mean difference of 16.9 bpm (95% CI: 5.4 to 28.3). The percentage of age-predicted maximum heart rate increased from 77 to 88%. The 6-min walk test performance showed a mean improvement of 219 m (95% CI: 167 to 272 m), from 271.4 ± 121 m to 490.7 ± 158.7 m.

#### Health-related quality of life outcomes

HeartQol scores improved from a mean of 0.8 ± 0.6 (physical) and 1.1 ± 0.7 (emotional) at baseline to 1.8 ± 0.6 and 2.4 ± 0.6, respectively, post-intervention (see Table 3). These changes correspond to mean differences of 1.0 (physical) and 1.3 (emotional).

**Table 3:** HeartQol score on the emotional and physical dimensions measured at baseline and 12 weeks post-surgery

	**Pretest ± SD* n* = 12**	**Posttest ± SD* n* = 11**	**MD (95% CI)**
Emotional dimensions	1.1 ± 0.7	2.4 ± 0.6	1.3 (0.9,1.6)
Physical dimension	0.8 ± 0.6	1.8 ± 0.6	1.0 (0.7,1.3)

*SD* Standard deviation, *MD* Mean difference, scale score 0 (poor) to 3 (excellent)

**Fig. 2:**
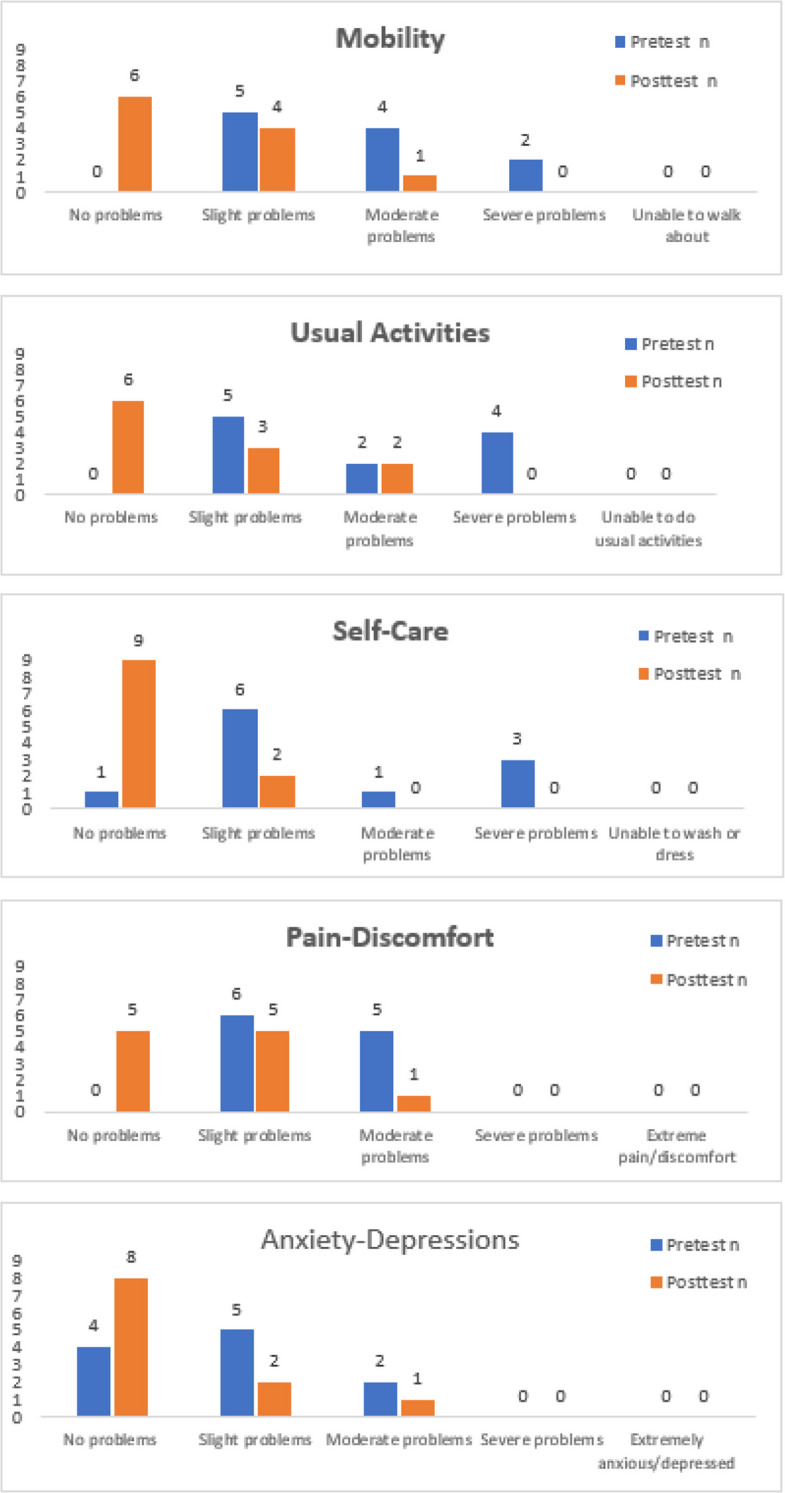
EQ-5D-5L scores on the five dimensions measured at baseline and 3 months post-surgery

The EQ-5D-5L VAS score increased from 56.6 ± 10.7 to 73.9 ± 12.0, with a mean change of 17.2 (95% CI 1.2–22.1). The EQ-5D-5L index rose from 0.61 ± 0.19 to 0.87 ± 0.12. EQ-5D-5L scores for the five dimensions are shown in Fig. 2.

## Discussion

This study demonstrates the feasibility of a 12-week interval training intervention for patients recovering from endocarditis following valve surgery. Early exercise after cardiac surgery has traditionally been considered relatively contraindicated in patients with systemic infection, including those with active infective endocarditis [32–34]. In the current study, adherence was satisfactory, adverse events were manageable, and functional capacity and health-related quality of life pointed towards possible improvements over the intervention period. However, due to the small sample size and the absence of a control group, these findings should be considered as preliminary, and caution is warranted when inferring treatment effects.

Most participants reported good acceptability regarding the timing of postoperative exercise initiation (mean 13 days after surgery), although some perceived the intervention to have started too late. The wide initiation window (4–36 days) reflects the heterogeneity of postoperative recovery in this patient group and highlights the challenges of standardising intervention timing in early-phase rehabilitation. These findings suggest that future trials may benefit from a more flexible approach, such as readiness-to-train criteria or stratification based on clinical stability, to balance individual recovery trajectories with protocol consistency.

Participant acceptability with smartwatch-based ECG and heart-rate monitoring varied. While real-time physiological feedback appeared to enhance engagement for some participants, others experienced challenges related to device usability or perceived limited added value. This variability underscores the importance of aligning digital monitoring tools with user needs and capabilities. In future studies, structured device familiarisation and clearer integration of wearable data into supervised exercise sessions may help optimise both usability and participant engagement.

Participant retention was high (92%), supporting the overall feasibility of the intervention. While this may partly reflect a motivated study population, high retention also suggests that the intervention and study procedures were manageable within the postoperative context. Future trials should nonetheless consider strategies to broaden inclusion and engagement to ensure generalisability across the wider endocarditis population.

The average adherence rate of 73% of the minimum expected sessions highlights some challenges in the post-surgical rehabilitation. Contributing factors that hindered engagement included varying postoperative recovery times, comorbidities, and interruptions such as illness or injury [35]. The challenges of a complex patient population highlight the importance of flexible and individualised programme timelines in future studies. To address such challenges, follow-up studies could incorporate adaptive scheduling, remote or hybrid exercise sessions, and closer monitoring of barriers to participation. Although only 11 participants completed the programme, the data provide valuable insights into recruitment, retention, adherence, and outcome variability, which can be used to estimate preliminary effect sizes and inform sample size calculations for a future fully powered randomised trial.

Beyond physiological improvements, the participants demonstrated notable enhancements in health-related quality of life, as evidenced by increased HeartQol and EQ-5D-5L scores. The observed improvements in the physical and emotional dimensions of HeartQol and EQ-5D-5L index scores could suggest that interval training may help mitigate the psychological burden associated with cardiac recovery, including anxiety and depression. These results are consistent with prior findings showing that structured exercise programmes improve both physical functioning and mental well-being in cardiac populations [36, 37]. These findings are particularly meaningful when viewed in the context of the considerable challenges faced by survivors of infective endocarditis, as documented in earlier studies. For instance, the CopenHeart IE survey reported persistent deficits in physical and mental health 1 year post-treatment, with patients scoring notably lower on physical and psychological health scales compared to the general population and to patients after heart valve surgery without IE [7]. Similarly, a study on survivors of left-sided native valve endocarditis highlighted prolonged physical symptoms and reduced quality of life, with 54% of patients reporting residual symptoms 1 year post-treatment. Furthermore, the presence of posttraumatic stress disorder (11%) in this population underlines the psychological challenges faced during recovery [5]. This may accentuate the need for further research into structured rehabilitation interventions, such as interval training, to determine their potential role in supporting physical and emotional recovery in this vulnerable patient population.

The intervention demonstrated an acceptable safety profile, with no serious adverse events such as myocardial infarction, stroke, infection, or re-sternotomy directly attributable to the training [9, 38]. However, adverse events in three participants underscore the importance of close monitoring in this vulnerable population. Post-pericardiotomy syndrome is one of the most common late postoperative complications following cardiac surgery. The condition is characterised by inflammation of the pericardium. It can result in symptoms such as chest pain, fever, and pericardial effusion, with an estimated incidence ranging from 10 to 30%, depending on the type of cardiac surgery performed [39–41]. Following a treadmill test in this study, its occurrence was likely unrelated to mobilisation and, more likely, part of the postoperative complication. The cases of post-pericardiotomy syndrome following a treadmill test, atrial fibrillation requiring medication adjustments, and a fall-related hip injury highlight the need for tailored safeguards. These include continuous ECG monitoring during exercise and personalised adjustments to training intensity to minimise potential risks. Notably, all participants who experienced complications completed the 12-week intervention, demonstrating that adverse events can be effectively managed within the structure of a supervised exercise programme. 

The average interval intensity (%APHRmax = 76.6%) should be interpreted in light of postoperative beta-blocker therapy. As beta-blockers blunt and delay the heart-rate response to exercise, and all participants were treated with metoprolol doses ranging from 50 to 150 mg during the intervention, pharmacological effects may have contributed to the lower age-predicted heart-rate response observed [42, 43].

The functional gains observed in this study should be interpreted with caution. Several factors likely contributed to these changes, including natural recovery and improvements in overall medical condition. VO₂ peak increased by 70% in our cohort, exceeding the 19–46% improvements reported in other studies of 12-week interval training in patients with various cardiac diseases [44, 45].

In comparison, a study from Denmark (the CopenHeart study) reported a 16% improvement in VO₂ peak among patients with endocarditis undergoing a 12-week rehabilitation programme, from 16.6 to 19.9 mL/kg/min [46]. The participants of the present study, however, started from a lower baseline (11.2 mL/kg/min) during the early postoperative phase (7–36 days after surgery), whereas CopenHeart initiated rehabilitation 4 weeks post-discharge, at a later stage of recovery with higher baseline values (~16 mL/kg/min). The lower starting point in our cohort likely explains the discrepancies from CopenHeart as well as the larger relative gains that were observed.

The tailored nature of our training protocol may also have improved adherence and adaptation, while individual variability and participant motivation likely played a role. Nevertheless, without a control group, it is impossible to determine how much of the improvement was due to the intervention itself versus natural recovery and regression to the mean. These results should therefore be interpreted as exploratory, providing preliminary indications of potential functional gains that can be achieved through early, tailored interval training.

Improvements observed across multiple parameters, including RER, VE, O₂ pulse, workload capacity, METs, 6-min walk distance, and %APHRmax (from 77 to 88%), may indicate broad-based aerobic and functional adaptations. While these findings are encouraging, they remain hypothesis-generating. They highlight the potential for greater improvements if early postoperative interval training is implemented, but the true effect compared with natural recovery remains uncertain.

**Fig. 3:**
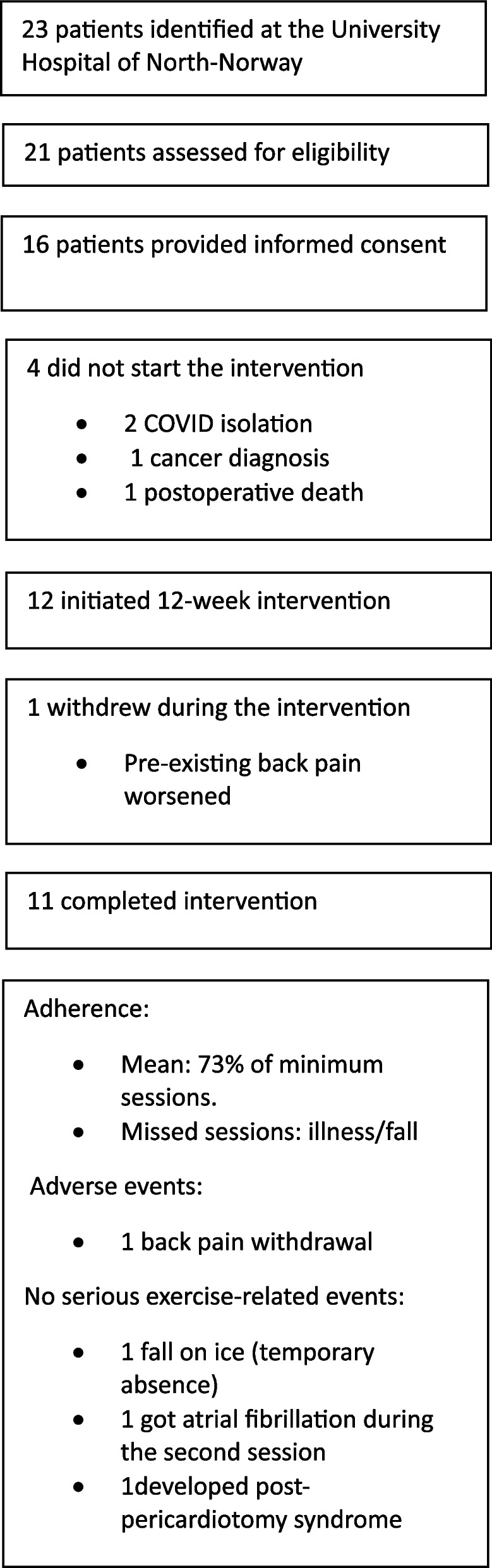
Participant flow and adherence in the feasibility study

Future studies should include a randomised controlled design, ideally comparing early interval training with standard care or later initiation of rehabilitation. Data from this feasibility study, including adherence and variability in functional responses, can inform sample size calculations for such studies. Additionally, future research should consider cost-effectiveness, as any structured programme must demonstrate added benefit beyond the natural recovery observed in this population (Fig. 3).

### Limitations and future directions

While this study provides valuable insights, several limitations have already been discussed. In addition, the reliance on %APHRmax as a measure of training intensity, although widely used, may not fully account for individual variability in heart rate responses. Future studies should include larger, randomised controlled trials to validate these findings and to examine the long-term sustainability of potential benefits. The use of more advanced physiological measures, such as heart rate variability or lactate thresholds, may also provide deeper insight into training adaptations.

## Conclusion

This study demonstrates that interval training, when implemented with appropriate safeguards and personalised approaches, is a feasible, acceptable, safe, and potentially effective intervention for patients recovering from endocarditis and heart valve surgery. Given the small sample size and recruitment bias, observed improvements in functional capacity, quality of life, and patient satisfaction should be viewed as exploratory and support the need for future controlled studies in this patient group.

## Supplementary Information

Supplementary Material 1. SPIRIT checklist

## Data Availability

The datasets are available from the corresponding author upon reasonable request.
