# Supplementary material for: Feasibility of early interval training in patients recovering from heart valve surgery due to infective endocarditis
Source: Pilot Feasibility Stud. 2026 May 12;12:93. doi: 10.1186/s40814-026-01830-w (PMC13335363; doi:10.1186/s40814-026-01830-w)
Supplement: Supplementary file 1 — Supplementary Material 1. SPIRIT checklist [file 40814_2026_1830_MOESM1_ESM.doc]

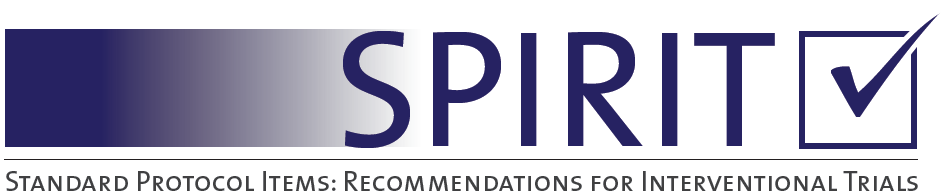


SPIRIT 2013 Checklist: Recommended items to address in a clinical trial protocol and related documents*

| Section/item | ItemNo | Description |
| --- | --- | --- |
| **Administrative information** | | |
| Title | 1 | Feasibility of early interval training in patients recovering from heart valve surgery due to infective endocarditis |
| Trial registration | 2a | Trial registration: Clinical Trials, ID NCT05703022. Registered: 25 November 2021, [http://www.ClinicalTrials.gov](http://www.ClinicalTrials.gov/) |
|  | 2b | N/A |
| Protocol version | 3 | N/A |
| Funding | 4 | This study was funded by the Norwegian Regional Health Authority (Helse Nord RHF, grant number HNF 1574-21) |
| Roles and responsibilities | 5a | Names, affiliations, and roles of protocol contributors |
|  | 5b | N/A |
|  | 5c | N/A |
|  | 5d | N/A |
| Introduction |  |  |
| Background and rationale | 6a | This study investigates the feasibility and acceptability of early-initiated interval training in patients recovering from heart valve surgery due to infective endocarditis (IE). Patients with IE often experience long-term physical and psychological impairments, yet are underrepresented in cardiac rehabilitation research. Previous work, including the CopenHeartIE trial (Rasmussen et al., 2022), showed positive effects of rehabilitation in this group but did not examine early or interval-based training. High-intensity interval training has proven effective in improving functional outcomes in other cardiac populations, but its safety and practicality post-IE remain unknown. This trial aims to fill that gap and inform the design of a full-scale study. |
|  | 6b | N/A |
| Objectives | 7 | The study aimed to assess the feasibility of early exercise training for patients recovering from heart valve surgery due to infective endocarditis, examining factors such as recruitment, retention, adherence, safety, benefits, and acceptability of the intervention. |
| Trial design | 8 | Feasibility study |
| Methods: Participants, interventions, and outcomes | | |
| Study setting | 9 | This was a single-centre, prospective feasibility study conducted at the University Hospital of North Norway (UNN), an academic tertiary care hospital in Tromsø, Norway. Patients were identified through the hospital’s patient administrative system following valve surgery for infective endocarditis. All data were collected in Norway. As this was a single-site study, a separate list of study sites is not applicable. |
| Eligibility criteria | 10 | **Inclusion criteria**:  (1) Patients presenting with confirmed infectious endocarditis aged > 18 years, with left-sided heart valve surgery and without arterial embolus; (2) Being residents of Northern Norway; (3) Willing and  able to give informed consent 4 - 21 days after the heart valve surgery.  **Exclusion criteria:**  (1) Patients who are hemodynamically or respiratory unstable, have a temperature > 38 ºC or have positive blood cultures; (2) Clinically significant severe concurrent medical conditions such as premorbid illnesses, other concurrent serious infections which could affect the safety or tolerability of the intervention; (3) Clinically significant concurrent musculoskeletal disorder or other concurrent disease or injury that may inhibit physical activity |
| Interventions | 11a | Participants participated in supervised aerobic interval training (4x4 models) for 30 to 40 minutes, 2 to 3 times a week. |
|  | 11b | Exercise sessions were discontinued if signs of functional deterioration were observed, including dyspnea, fever, arrhythmia, or worsening heart failure. |
|  | 11c | The intervention was individualised to promote adherence based on each patient’s clinical status and functional capacity. Adjustments to the interval training protocol, such as the intensity and duration of intervals, were made to ensure safety and tolerability. Participants’ heart rates were continuously monitored using a validated smartwatch with electrocardiography (ECG) functionality to guide intensity and enhance safety during sessions. Adherence was monitored through attendance records kept by supervising physiotherapists and smartwatch training logs. Equipment (e.g., treadmill or stationary bike) was selected based on patient preference and functional assessment to increase engagement and compliance. All participants received standard postoperative beta-blocker therapy, and clinical monitoring was performed to manage potential complications that could interfere with exercise participation. |
|  | 11d | N/A |
| Outcomes | 12 | The primary outcome was feasibility, assessed through recruitment rate (proportion of eligible patients who consented), retention rate (proportion completing the intervention), and adherence (percentage of scheduled sessions attended). These were summarised as proportions and descriptive statistics at the end of the 12-week intervention. This outcome is crucial to determine the practicality of delivering early interval training in this patient group.  Functional outcomes included:  Functional capacity, measured by submaximal VO₂ (ml/kg/min), workload (W), and six-minute walk distance (meters); the analysis metric was change from baseline to 12 weeks, reported as means and 95% confidence intervals.  Health-related quality of life was assessed by HeartQol (physical and emotional dimensions) and EQ-5D-5L (index and VAS); changes were analysed from baseline to 12 weeks, using mean values and 95% confidence intervals.  These outcomes were selected for their clinical relevance: functional capacity reflects recovery and long-term prognosis, while quality of life measures the patient-perceived benefit of rehabilitation. Monitoring for adverse events and reasons for dropout served as proxies for harm assessment. |
| Participant timeline | 13 | Participants were enrolled postoperatively when hemodynamically and respiratory stable. The intervention (interval training) started 7 and 36 days after surgery and lasted 12 weeks. Baseline assessments were performed before the intervention, including a submaximal VO2 test, a 6-minute walk test, and quality of life questionnaires (HeartQoL and EQ-5D-5L). The same assessments were repeated after the intervention at 12 weeks. Compliance and exercise data (session frequency, duration, intensity) were recorded throughout the intervention. |
| Sample size | 14 | The estimated sample size was based on feasibility study guidelines rather than formal power calculations. According to recommendations in the feasibility literature, a sample size of 12 to 30 participants is generally sufficient to assess key feasibility outcomes, such as recruitment, retention, and adherence, with reasonable precision for informing the design of a future full-scale trial. We pragmatically set a recruitment target of up to 20 participants, aiming for approximately 12 to complete the intervention. This range was considered adequate to identify significant barriers to participation and implementation, and to gather preliminary data on the intervention’s acceptability and delivery. |
| Recruitment | 15 | To support adequate participant enrolment and allow sufficient time for postoperative recovery, we implemented a broad inclusion window, initiating the intervention between 7 and 36 days after surgery once participants were hemodynamically and respiratory stable. This flexible approach increased the likelihood of patient eligibility and willingness to participate. Additionally, we collaborated closely with local hospitals and municipal healthcare services to ensure they had the necessary knowledge and resources to deliver the intervention locally, thereby reducing travel barriers and enhancing accessibility. |
| **Methods: Assignment of interventions (for controlled trials)** | | |
| Allocation: |  |  |
| Sequence generation | 16a | N/A |
| Allocation concealment mechanism | 16b | N/A |
| Implementation | 16c | N/A |
| Blinding (masking) | 17a | N/A |
|  | 17b | N/A |
| **Methods: Data collection, management, and analysis** | | |
| Data collection methods | 18a | Baseline and outcome data were collected using validated instruments, including HeartQoL, EQ-5D-5L, Borg RPE scale, 6-minute walk test, and submaximal VO₂ testing on a treadmill. Exercise data (e.g., intensity, duration, and heart rate) were logged via a specially designed log questionnaire. |
|  | 18b | Participants who consented to the study (n=16) received regular information and encouragement to support their continued engagement. The study was conducted during the COVID-19 pandemic, and four participants withdrew due to COVID-19 infection or postoperative complications. An additional participant discontinued the intervention because of back pain. Although no formal dropout analysis was conducted, baseline data were retained for all participants to support the evaluation of feasibility outcomes. |
| Data management | 19 | Data were entered into a secure electronic UNN database, coded, and stored following local data protection guidelines (POV UNN). |
| Statistical methods | 20a | Statistical methods for analysing primary and secondary outcomes. Reference to where other details of the statistical analysis plan can be found, if not in the protocol  Descriptive statistics were applied to analyse feasibility outcomes (e.g., recruitment rate, adherence, and retention) and exploratory outcomes (e.g., changes in VO₂ peak and health-related quality of life). Continuous variables are presented as means with standard deviations (SD), and mean changes from baseline to follow-up are reported with 95% confidence intervals (CI). All statistical analyses were conducted using IBM SPSS Statistics, version 29.0.1.0. |
|  | 20b | N/A |
|  | 20c | N/A |
| **Methods: Monitoring** | | |
| Data monitoring | 21a | Composition of data monitoring committee (DMC); summary of its role and reporting structure; statement of whether it is independent from the sponsor and competing interests; and reference to where further details about its charter can be found, if not in the protocol. Alternatively, an explanation of why a DMC is not needed |
|  | 21b | Description of any interim analyses and stopping guidelines, including who will have access to these interim results and make the final decision to terminate the trial |
| Harms | 22 | Plans for collecting, assessing, reporting, and managing solicited and spontaneously reported adverse events and other unintended effects of trial interventions or trial conduct |
| Auditing | 23 | Frequency and procedures for auditing trial conduct, if any, and whether the process will be independent from investigators and the sponsor |
| Ethics and dissemination | | |
| Research ethics approval | 24 | The study was approved by the Regional Committees for Medical and Health Research Ethics in Norway (Project number 228905) |
| Protocol amendments | 25 | All necessary protocol modifications, such as changes to eligibility criteria, outcomes, or analytical approaches, will be reported to the Regional Committees for Medical and Health Research Ethics (REK) in Norway by national regulations. |
| Consent or assent | 26a | N/A |
|  | 26b | N/A |
| Confidentiality | 27 | Personal information about potential and enrolled participants was collected and stored following guidelines approved by the Data Protection Authority at the University of North Norway (Project number: 02752). All identifiable data were securely stored in encrypted and password-protected databases, accessible only to authorised study personnel. Each participant was assigned a unique study ID, and all analyses were performed on de-identified data. Personal data were not shared outside the research team and will be retained only as long as necessary for study purposes, after which they will be securely deleted. |
| Declaration of interests | 28 | N/A |
| Access to data | 29 | N/A |
| Ancillary and post-trial care | 30 | N/A |
| Dissemination policy | 31a | The investigators plan to disseminate the trial results through scientific publications in peer-reviewed journals and presentations at national and international professional conferences. In addition, the findings will be shared with relevant clinical and academic communities through targeted dissemination within professional networks and specialist groups. There are no publication restrictions. Results will also be made available to participants and healthcare providers involved in the study through summary reports or professional briefings. |
|  | 31b | N/A |
|  | 31c | N/A |
| Appendices |  |  |
| Informed consent materials | 32 | N/A |
| Biological specimens | 33 | N/A |

*It is strongly recommended that this checklist be read in conjunction with the SPIRIT 2013 Explanation & Elaboration for important clarification on the items. Amendments to the protocol should be tracked and dated. The SPIRIT checklist is copyrighted by the SPIRIT Group under the Creative Commons “[Attribution-NonCommercial-NoDerivs 3.0 Unported](http://www.creativecommons.org/licenses/by-nc-nd/3.0/)” license.
